# Current Guidelines for Blood Conservation in Cardiac Surgery

**Published:** 2009-06

**Authors:** Yatin Mehta, Jeetendra Sharma

**Affiliations:** * Director, Deptt. of Anaesthesiology & Critical Care; ** Consultant Anaesthesiology and Critical Care

Cardiac surgery is associated with excessive bleeding as compared to non-cardiovascular surgery. In many situations excessive bleeding is expected but unexpected bleeding may pose problem during and, or after surgery. Studies have found certain predictors for bleeding like, increased age, emergency surgery, low body surface area, prolonged cardiopulmonary bypass (CPB) time > 150 minutes, combined intracardiac and bypass surgery, number of bypass grafts (> 4), reoperative surgery and preoperative antiplatelets agent. Between 3% to 14% of patients with significant bleeding require re-exploration.^1^ A surgically correctable source of bleeding is found in 50% - 67% of patients.^2^ Bleeding and surgical re-exploration are both independent predictors of an adverse outcome.^2^ Thus reducing this bleeding is a desirable clinical goal. Although the benefits of red cell blood transfusions for managing life-threatening bleeding are clear but allogenic blood product transfusion is consistently associated with several adverse effects.^3^

Liberal use of red blood cells transfusion is associated with increased nosocomial infection and mortality in critically ill patients.^4^ Blood transfusion during or after coronary artery bypass graft (CABG) surgery has been shown to be associated with increased long-term mortality.^5^

Current blood conservation practice guidelines suggest that institutions strategy should start with preoperative evaluation to identify high risk patients and appropriate management of antiplatelet therapy. In all planned surgery thienopyridines (clopidogrel) should be stopped 5 to 7 days prior to surgery except in patients with drug eluting stents, in whom sudden withdrawal of antiplatelets can result in sudden stent thrombosis. Aspirin should only be discontinued in purely elective cases without acute coronary syndrome. The addition of clopidogrel to aspirin increases postoperative haemorrhage. Yende and Wunderink demonstrated that aspirin and clopidogrel increase postoperative bleeding sevenfold.^6^ Heparin is an integral component of therapy for acute coronary events. Patients receiving low molecular weight heparin (LMWH) within 12 hours of cardiac surgery have significantly greater blood loss and increased blood transfusion compared with patients receiving intravenous heparin or a dose of LMWH more than 12 hours before operation. Unfractionated heparin is a notable exception in that it is the only agent that may be discontinued shortly before operation or not at all.

Evidence for transfusion trigger recommends use of haemoglobin level and platelets count for red cells and platelets transfusion respectively. More advanced measurements such as whole body oxygen-carrying capacity, oxygen consumption, oxygen extraction ratios, and oxygen delivery provide more accurate means to estimate the need for red blood cell transfusions.^7^ For postoperative patients and off CPB surgeries guidelines recommend below 7-gm/dl haemoglobin level as transfusion trigger for red cells transfusion except in evident cardiac or non-cardiac end organ ischaemia where desirable haemoglobin level is 10 gm/dl. Patients having haemoglobin level more than 10 gm/dl should not be transfused because there is risk related to transfusion without favourable improvement in oxygen transportation. Transfusion trigger is further reduced to 6 gm/dl on CPB with moderate hypothermia except in patients with history of cerebrovascular disease, diabetes mellitus, and carotid stenosis.^8^ However extracorporeal circulation is associated with a heavy fluid load that may significantly decrease haemoglobin concentration due to haemodilution. Thus, considering haemoglobin alone may be an inaccurate method of replacing red cell volume loss on CPB. Slight et al found that considering haemoglobin concentration alone may significantly overestimate the requirement for red cell transfusion in elective cardiac surgery patients. Patients transfused as per the red cell volume-based guideline received significantly less red cells with no associated difference in clinical outcome.^9^

Intraoperative techniques in blood conservation cannot be underemphasized. Meticulous haemostasis and operative technique can play an important role in reducing blood loss. Acute normovolaemic haemodilution (ANH) has not only been shown to be more cost effective than preoperative autologous donation but also is not limited by time restraints preoperatively. The strategy behind ANH is to lower the red blood cell mass loss during surgery while preserving clotting factors. However, the efficacy of ANH is controversial. Segal et at showed that ANH was only moderately effective in reducing transfusions by 10% or 1 to 2 units less than the control group.^10^ The recent ability to separate blood collected in the operating room into individual products, such as red blood cells, platelet rich plasma, and platelet poor plasma, thus allowing these products to be returned individually as indicated, may suggest that the role of ANH be revisited.^11^

To limit blood transfusions, The Society of Thoracic Surgeons(STS) and The Society of Cardiovascular Anesthesiologists(SCA) guidelines recommend use of aprotinin and lysine analogues epsilon am inocaproic acid (EACA) and tranexamic acid (Cyclokapron) in high risk patients. Aprotinin (Trasylol) and the lysine analogues have very different modes and scope of action but ultimately inhibit fibrinolysis by limiting the action of plasmin. A metaanalysis on aprotinin concluded significant reduction in blood transfusion in patients undergoing CABG, redo CABG and valve replacement.^12^ Though aprotinin reduces blood transfusion significantly but blood conservation using antilibrinolytics randomized trial(BART) investigators found more mortality in aprotinin treated patients in high risk cardiac surgery cf. tranexamic acid.^13^

Factor VIIa is recommended for intractable bleeding, unresponsive to usual hemostatics and non surgical means.^8^ We successfully used it for reredo cardiac surgery with intractable bleeding.^14^ Likewise recombinant erythropoietin can be used in patients of, autologous preoperative blood donation and anaemia, however evidence is conflicting in different trials.

Some intervention and modification during CPB are useful in blood conservation. Open reservoir membrane oxygenator system during CPB may reduce blood utilization and improve safety. Similarly activated clotting time (ACT) guided heparin dosing during prolonged CPB not only reduce blood transfusion but also haemostatic system activation, and platelets and proteins consumption as compare to fixed dose heparin supplements. Retrograde autologous priming of the CPB circuit, intraoperative autotransfusion, either with blood directly from cardiotomy suction or recycled using a cell-saving device, shortly after the completion of CPB, salvage of pump blood, either administered without washing or after washing with a cell-saving device should be used for blood conservation.^8^

Lastly, one cannot ignore importance ofa multimodality approach involving multiple stakeholders, institutional support and enforceable transfusion algorithms supplemented with point-of-care testing to prevent blood loss and blood transfusion.^8^

This becomes particularly important in a developing country like India with limited resources, variable quality of blood banking and surgical expertise with exponential growth of cardiac surgical centres. The role of anaesthesiologist is vital for implementing evidence based transfusion practices in cardiac surgery.
